# Management of enterocutaneous (jejunocutaneous) fistulas with NBCA glue injection: A case report

**DOI:** 10.1016/j.radcr.2025.04.038

**Published:** 2025-05-12

**Authors:** Paria Bolourinejad, Ghazal Zandieh, Pardis Tavallaeinejad, Mohammad Saleh Jafarpisheh

**Affiliations:** a Student Research Committee, School of Medicine, Isfahan University of Medical Sciences, Isfahan, Iran; b School of Medicine, Isfahan University of Medical Sciences, Isfahan, Iran; c Department of Radiology, Isfahan University of Medical Sciences, Isfahan, Iran

**Keywords:** Enterocutaneous fistula, n-Butyl cyanoacrylate glue, Minimally invasive treatment, Crohn's Disease, Fistula closure, Patient-centered care, Jejunocutaneous fistula

## Abstract

This case report highlights a minimally invasive approach to the management of enterocutaneous fistulas (ECFs), specifically jejunocutaneous fistulas (JCFs), using n-butyl cyanoacrylate (NBCA) glue. The patient, a 77-year-old female with Crohn's disease, presented with abdominal pain, fever, chills, and persistent discharge from the JCF site. Conventional treatment options for ECFs, ranging from conservative management to surgical intervention, are often associated with morbidity and prolonged recovery periods. This case demonstrates the successful closure of a JCF using NBCA glue injection, resulting in complete symptom resolution and sustained fistula closure at 18 months follow-up. The application of NBCA glue represents a promising alternative to traditional approaches, offering reduced procedural risks and shorter recovery times. This report highlights the role of minimally invasive techniques in the management of ECFs and highlights the need for further studies to establish standardized protocols and expand the clinical use of this approach.

## Introduction

Enterocutaneous fistulas (ECFs) are abnormal connections between the intestinal lumen and the skin, often resulting in malnutrition, sepsis, and delayed wound healing. Among these, jejunocutaneous fistulas (JCFs) are particularly challenging, commonly arising as postoperative complications in Crohn’s disease [[1], [2], [3]].

Conservative management including bowel rest, total parenteral nutrition (TPN), and infection control can be effective but often fails in cases with high-output fistulas or underlying sepsis, necessitating surgical intervention. However, surgery carries significant risks, including morbidity, prolonged recovery, and recurrence [4].

Minimally invasive techniques, such as n-butyl cyanoacrylate (NBCA) glue injection, have emerged as promising alternatives. NBCA glue rapidly polymerizes upon contact with moisture, effectively sealing fistula tracts while minimizing procedural risks [[5], [6], [7]]. This report presents a case of successful JCF closure using NBCA glue, highlighting its potential as a safe and effective nonoperative treatment option.

### Case presentation

This report presents the case of a 77-year-old female with a 4-year history of Crohn's disease complicated by recurrent jejunocutaneous fistula (JCF) formation. The fistula developed following multiple episodes of colonic diverticulitis and subsequent abscess formation, with persistent purulent and bloody discharge (approximately 5 mL/day). Her medical history was notable for diabetes mellitus, managed with anti-diabetic medications. The patient’s Crohn's disease had been managed with Mesalamine suppositories, which were discontinued after 10 days due to rectal bleeding. She was subsequently prescribed Azathioprine 50 mg twice daily and Prednisolone 5 mg twice daily. Additionally, she received multiple courses of antibiotics, including Oxacillin, Ciprofloxacin, Clindamycin, Metronidazole, and Levofloxacin, over 4 months. Initial management involved percutaneous drainage of the abscess and the JCF, which provided temporary relief. However, the patient experienced biweekly recurrences characterized by worsening abdominal pain, fever, chills, and increased purulent and bloody discharge from the JCF site (up to 20 mL/day). Despite these interventions, the fistula persisted, prompting consideration of alternative management strategies.

**Fig. 1:**
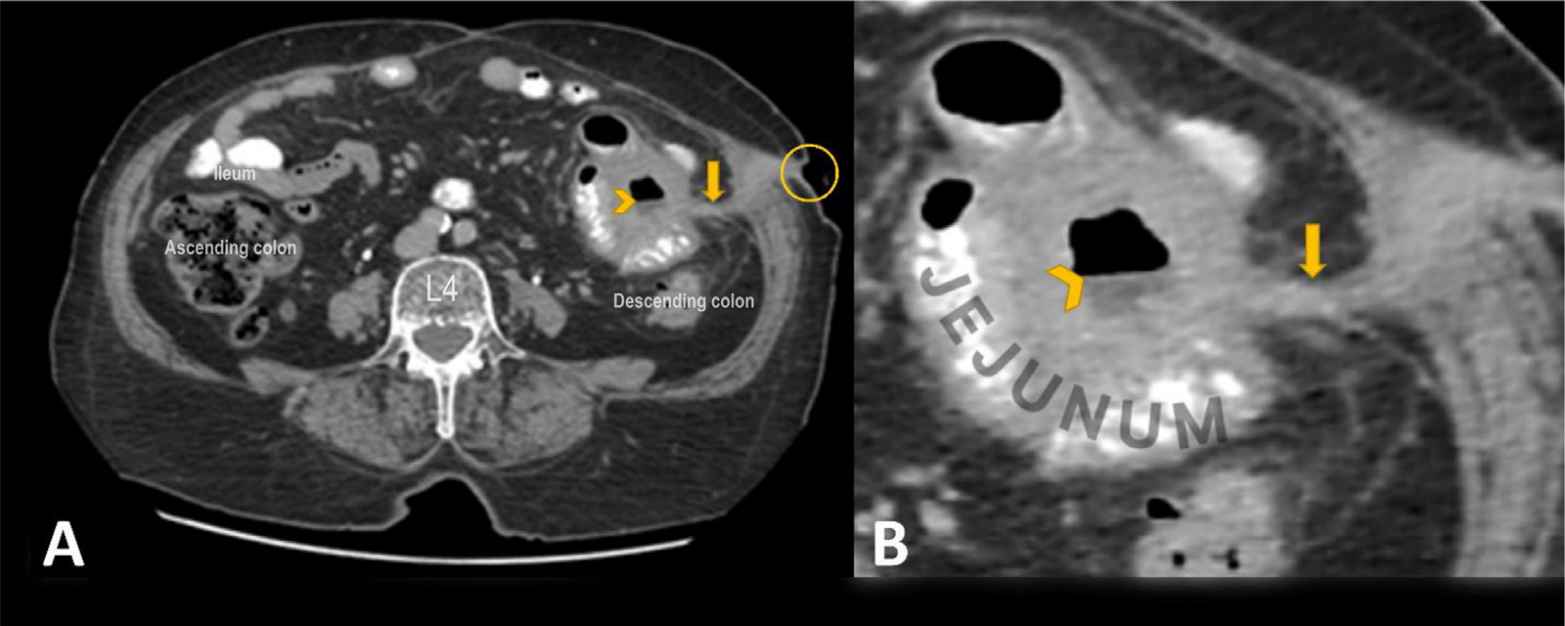
(A) Axial abdominopelvic CT scan with intravenous (IV) and oral (PO) contrast showing a 55-mm fistulous tract (arrow) connecting peritoneal collections and the anterior abdominal wall in the left lower quadrant (LLQ), adjacent to the jejunoileal loops (the skin orifice is marked by a circle). A peritoneal collection measuring 22 × 16 mm is also noted (arrowhead). (B) Magnified view of panel A highlighting the fistulous tract and associated findings.

Imaging studies revealed a 55-mm fistulous tract extending between peritoneal collections and the anterior abdominal wall in the left lower quadrant (LLQ), adjacent to the jejunoileal loops. Additionally, 2 peritoneal collections measuring 22 × 16 mm and 23 × 13 mm were identified. These collections which have air-fluid levels caused thickness of the collection wall, adherence to surrounding jejunum loops, and unilateral thickening in intestinal loops due to their chronicity (Fig. 1). Given the failure of conservative management and the patient's refusal to undergo surgical intervention, an approach utilizing n-butyl cyanoacrylate (NBCA) glue injection was proposed as a minimally invasive alternative for fistula closure. After a thorough multidisciplinary consultation and a detailed discussion of the experimental nature of this technique, including its potential benefits, risks, and alternatives, the patient provided informed written consent to proceed with the procedure.

### Management and outcome

The procedure was performed in a tertiary referral hospital in Isfahan, Iran, under the supervision of an interventional radiologist in a dedicated angiography suite. The patient was positioned supine to ensure optimal access to the jejunocutaneous fistula (JCF) site, and the puncture site was cleaned and draped in a sterile manner. Local anesthesia was administered using lidocaine 2% for patient comfort during the procedure.

**Fig. 2:**
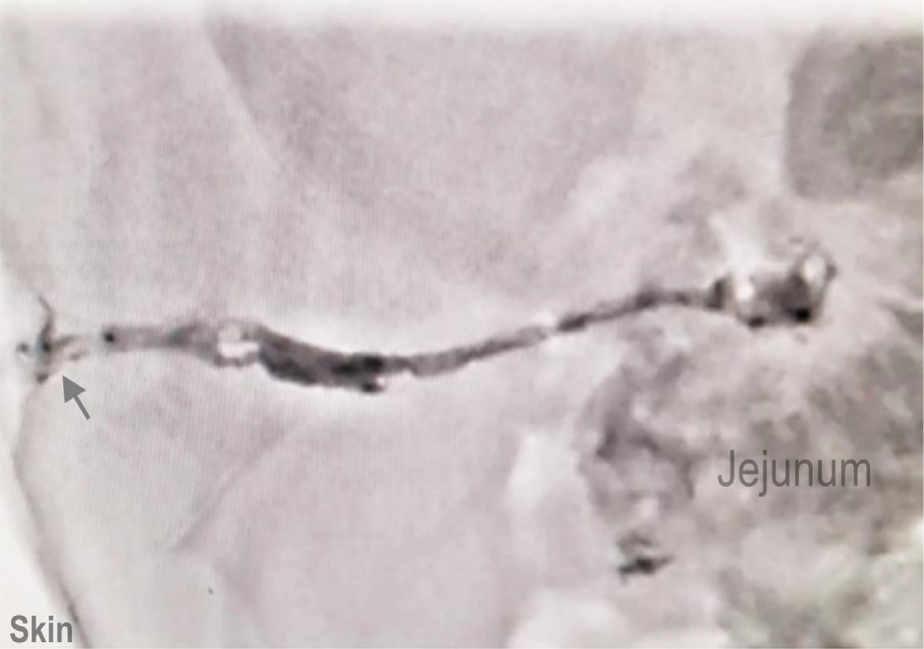
Under fluoroscopic guidance, fistulography was performed to delineate the anatomy of the fistula tract. A gray cannula was used to access the skin orifice. Contrast injection revealed a 55-mm-long tract connecting the jejunal loops to the anterior abdominal wall. A microwire was guided through the cannula sheet into the fistula. After reaching the clone, the cannula sheet was withdrawn. Using this guidance, a Mastro microcatheter (inner diameter: 0.018 F; outer diameter: 2.4 F) was percutaneously advanced through the fistulous tract into the jejunal loop, followed by the removal of the microcatheter. This fluoroscopic image showing the injection 1:1 mixture of n-butyl cyanoacrylate (NBCA) glue (Histoacryl; B. Braun, Tuttlingen, Germany) and a radiopaque agent (Lipiodol Ultra-Fluide; Guerbet, Aulnay-sous-Bois, France) into the jejunocutaneous fistula (JCF). The glue is visualized as it fills the fistulous tract, sealing the connection between the jejunal loop and the skin surface.

Under fluoroscopic guidance, a fistulography was performed to delineate the anatomy of the fistula tract. Using a gray cannula, the skin orifice of the fistula was selected, and 20 cc of 50% contrast was injected. The contrast agent revealed a 55-mm-long tract connecting the jejunal loops to the anterior abdominal wall, providing a precise roadmap for catheter placement, and there was no contrast extravasation into the peritoneal cavity. After seeing the track pathway, a microwire was guided through the cannula sheet into the fistula. After reaching the clone, the cannula sheet was withdrawn. Using this guidance, a Mastro microcatheter (inner diameter: 0.018 F; outer diameter: 2.4 F) was percutaneously advanced through the fistulous tract into the jejunal loop (Fig. 2). If glue had been injected without microcatheter, it would have polymerized before reaching the jejunal loops. Therefore, due to long fistula tract and its connection to intestinal loops, we decided to perform the procedure with microcatheter. Finally, the microwire was removed from the microcatheter. The treatment involved injecting a 1:1 mixture of n-butyl cyanoacrylate (NBCA) glue (Histoacryl; B. Braun, Tuttlingen, Germany) and a radiopaque agent (Lipiodol Ultra-Fluide; Guerbet, Aulnay-sous-Bois, France). This combination was chosen for its rapid polymerization, radiopacity, and ability to precisely seal the fistula under fluoroscopic visualization. The glue mixture was delivered retrograde through the microcatheter to completely fill and seal the tract from the jejunal loop to the skin surface. The entire procedure was completed within 10 minutes.

The patient was closely monitored for potential adverse events such as embolization, allergic reaction, or tissue irritation during a 4-hour observation period. No immediate or delayed complications, including tissue inflammation, were observed. The patient reported significant symptom relief and was discharged in stable condition with instructions for follow-up care. At the 18-month follow-up, the patient remained asymptomatic, with imaging confirming complete closure of the JCF and no recurrence of fistula-related symptoms. This outcome highlights the efficacy and safety of NBCA glue injection as a minimally invasive alternative for managing complex JCFs, particularly in patients who are not surgical candidates.

**Fig. 3:**
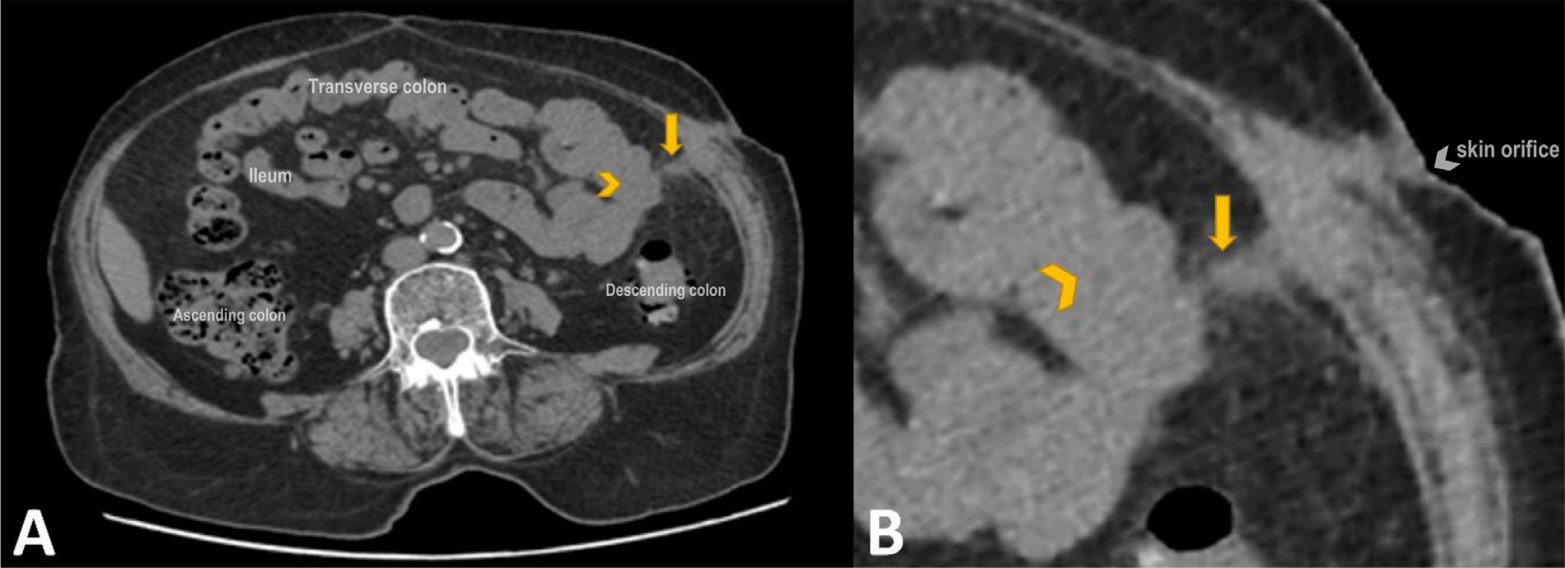
(A) Axial abdominopelvic CT scan (without contrast) obtained after the intervention, showing a fibrotic fistulous tract (arrow) with no evidence of residual peritoneal collections (arrowhead). (B) Magnified view of panel A confirming the successful closure of the fistula. the fistula tract appeared retracted and shorter compared to its preprocedural state, with beading in the tract.

**Fig. 4:**
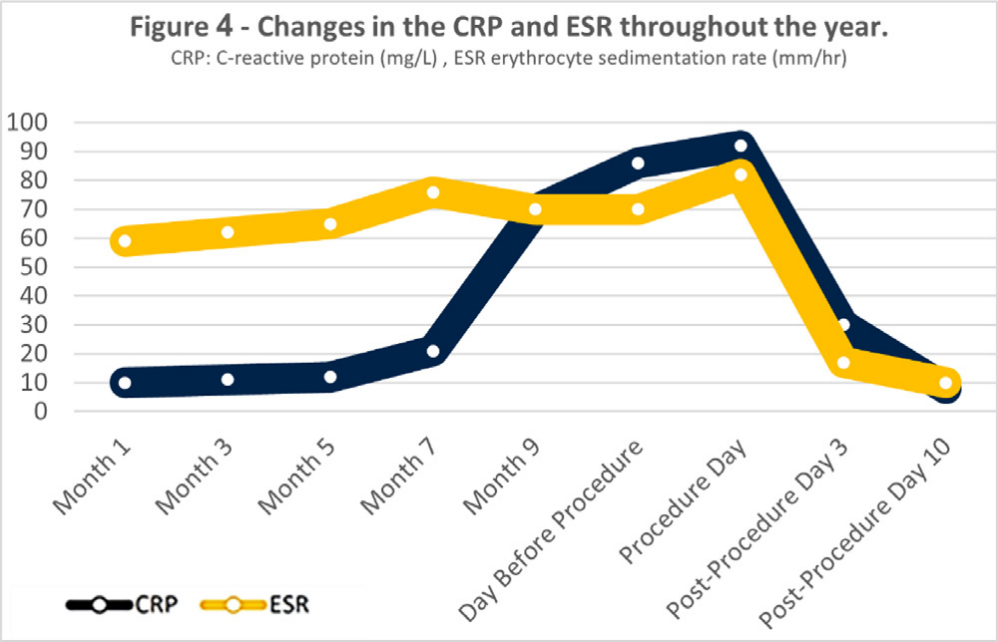
Trends in C-reactive protein (CRP) and erythrocyte sedimentation rate (ESR) levels over 1 year, demonstrating a significant decline following the intervention, consistent with the resolution of inflammation and successful management of the jejunocutaneous fistula (JCF).

The patient experienced complete resolution of clinical symptoms after the intervention. Tubography performed on day 3 postprocedure confirmed successful closure of the fistulous tract, with no evidence of contrast leakage. Subsequent follow-up imaging, including abdominopelvic CT scans, demonstrated fibrosis at the fistula site without any residual peritoneal collections or new fistula formation. Additionally, the fistula tract appeared retracted and shorter compared with its preprocedure state (Fig. 3). Laboratory markers of inflammation, including C-reactive protein (CRP) and erythrocyte sedimentation rate (ESR), showed significant improvement, correlating with the patient’s clinical recovery (Fig. 4). By 18 months’ postprocedure, the patient remained symptom-free, with no recurrence of the fistula or related complications, highlighting the long-term efficacy of the intervention.

The patient resumed a normal diet, regained weight, and experienced an improved quality of life, though Crohn's flares still occur. For future follow-ups, no imaging is needed unless there is recurrent discharge, abdominal pain, fever, or other related symptoms.

This case underscores the potential of n-butyl cyanoacrylate (NBCA) glue as a minimally invasive and effective treatment option for complex jejunocutaneous fistulas, particularly in patients where surgical interventions are not feasible. Further studies are warranted to standardize this technique and evaluate its broader applicability in fistula management.

## Discussion

The use of n-butyl cyanoacrylate (NBCA) glue for the closure of a jejunocutaneous fistula (JCF) in this patient with Crohn's disease represents an innovative, minimally invasive approach to managing complex enterocutaneous fistulas (ECFs). This technique aligns with the growing emphasis on reducing procedural risks and morbidity while improving patient outcomes [8]. Our case highlights the long-term efficacy of NBCA glue in achieving durable fistula closure, with sustained results observed at 18 months’ postprocedure.

Tissue adhesives are classified into biomimetic, synthetic, natural protein-based, and hybrid. N-butyl 2-cyanoacrylate (n-BCA), octyl cyanoacrylate (OCA), and 2-octyl cyanoacrylate (2-OCA) are the different types of cyanoacrylate glue. Cyano helps in binding protein, while acrylate helps in polymerization, and combination of both causes tissue healing. The term "cyanoacrylate" is based on the cyano, ethylene, and various alkyl groups [9]. NBCA glue has demonstrated versatility in managing various gastrointestinal fistulas beyond JCFs. In 1 case, a pouch-cutaneous fistula following restorative proctocolectomy was successfully treated with cyanoacrylate glue injection, achieving complete closure without recurrence at 5 months’ follow-up. Similarly, NBCA glue has been used in the management of pancreatic-cutaneous fistulas, ileal pouch-anal anastomotic leaks, and other postoperative complications [10,11]. These cases, along with our report, underscore the utility of NBCA glue as a safe and effective alternative to surgical intervention, particularly in patients with high surgical risks or those who refuse surgery.

Fibrin glue is another agent in the management of GI fistulae and has been found to achieve nonoperative closure of low-output as well as high-output GI fistulae. The main disadvantage of using gelfoam and fibrin glue as embolic agents are their weaker tensile and adhesive strength compared to cyanoacrylates, and this fact might explain the higher failure rate associated with the former ones. Furthermore, fibrin glue is an animal protein with inherent risks of allergy and prion disease transmission and may also be broken down by enzyme-rich fluid secreted in gastric or pancreatic fistulae, making it unsuitable for this role. Cyanoacrylates are also more cost-effective than fibrin glue [12].

The application of NBCA glue in our case involved a 1:1 mixture of NBCA and Lipiodol Ultra-Fluid, delivered retrograde through a microcatheter under fluoroscopic guidance. This approach allowed precise visualization and sealing of the fistulous tract, leading to rapid symptom resolution and a successful outcome. This mixture was chosen due to the long and tortuous nature of the fistula for its lower viscosity and to prevent rapid polymerization.

The minimally invasive nature of this technique offers significant advantages over conventional surgical management, which is associated with prolonged recovery, higher morbidity, and recurrence risks. As such, NBCA glue holds promise as a first-line treatment for selected cases of low-output ECFs.

However, this technique is not without limitations. Complications such as inadvertent sealing of the bowel lumen may arise if the catheter is improperly positioned or if injection pressure is too high. Proper bowel dilation with saline and contrast prior to glue injection is essential to minimize these risks. Incomplete sealing of the fistula’s enteric orifice can lead to abscess formation, necessitating careful procedural planning and execution. Additionally, multiple sessions may be required for complete fistula closure in certain cases, and late complications such as intra-abdominal adhesions should be monitored. Another challenge of this approach is that the polymerized glue is not aligned continuously, which can cause microabscess formation due to a blocked outlet. Since this patient has been taking antibiotics, this risk is reduced. Additionally, intestinal peristalsis can push the glue out of the tract. Due to the length of the tract, this risk is relatively low in this patient.

Patient selection is critical—low-output ECFs with narrow fistulous tracts are ideal candidates, whereas patients with extensive bowel wall disruption or wide tracts may be less suitable. Further experience and data are required to refine these criteria [13].

The potential of NBCA glue to expand beyond vascular embolization to nonvascular applications reflects its adaptability and growing importance in minimally invasive therapies. The high success rates and minimal morbidity reported in the literature make NBCA glue an appealing alternative to surgery for refractory gastrointestinal fistulas [12]. Our case further supports this technique’s effectiveness in managing JCFs, highlighting its role in bridging the gap between conservative and surgical treatments.

Future research should focus on optimizing procedural protocols, including catheter positioning, glue formulations, and methods to prevent complications. Comparative studies are also needed to evaluate the long-term outcomes of NBCA glue versus surgical and other minimally invasive approaches. Additionally, identifying patient-specific factors that predict success or failure will help establish standardized selection criteria, ensuring optimal outcomes for this promising technique.

The case underscores the potential of NBCA glue embolization as a viable, less invasive alternative to traditional surgical approaches in managing enterocutaneous fistulas with peritoneal collections. This method offers a reduction in surgery-related morbidity and mortality, alongside shorter hospital stays, highlighting its utility as a treatment option in the complex therapeutic landscape of ECF management.

## Declaration

The tents of the Declaration of Helsinki were followed.

## Patient consent

The patient was informed about the procedure and its potential complications. Informed consent was obtained before proceeding with the procedure. Additionally, the patient consented to the use of her images and other relevant clinical information to be included in this article.
